# The Role of Monocyte Distribution Width in the Early Prediction of Sepsis in Patients Undergoing Cardiovascular Surgery: A Cross-Sectional Study

**DOI:** 10.3390/medicina60091558

**Published:** 2024-09-23

**Authors:** Abdullah Özer, Sercan Tak, Hüseyin Demirtaş, Alperen Kutay Yıldırım, Elif Şimşek, Gürsel Levent Oktar, Zühre Kaya

**Affiliations:** 1 Department of Cardiovascular Surgery, Gazi University Faculty of Medicine, 06500 Ankara, Turkey; abdullahozer@gazi.edu.tr (A.Ö.); sercantak@gazi.edu.tr (S.T.); hdemirtas@gazi.edu.tr (H.D.); akyildirim@gazi.edu.tr (A.K.Y.); elifsimsek@gazi.edu.tr (E.Ş.); gloktar@gazi.edu.tr (G.L.O.); 2 Department of Pediatric Hematology, Gazi University Faculty of Medicine, 06500 Ankara, Turkey

**Keywords:** monocyte distribution width, cardiovascular surgery, sepsis

## Abstract

*Background and Objectives:* This is the first study to examine the role of monocyte distribution width (MDW) in predicting sepsis after cardiovascular surgery. *Methods:* This study included 43 consecutive patients who had undergone cardiovascular surgery between July 2021 and July 2022. All patients were examined at the following three time points (TPs): preoperative period (TP1), postoperative at 24 h (TP2), and discharge (TP3). SOFA score, leukocyte count, neutrophil-to-lymphocyte ratio (NLR), MDW, C-reactive protein (CRP), and procalcitonin (PCT) levels were tested at each TPs. The Sepsis-3 criteria were used to diagnose patients with sepsis. *Results:* The mean values of all variables (leukocyte count, NLR, MDW, CRP, and PCT levels) were significantly higher at TP2 and TP3 than at TP1 (*p* < 0.05). All these values were significantly higher at TP2 than at TP3 (*p* < 0.05). Patients with sepsis had significantly higher mean values for leukocyte count, NLR, MDW, CRP, and PCT levels than those without sepsis (*p* < 0.05). There was a significant correlation between MDW and inflammatory markers (CRP, PCT, and NLR) during the three time periods (*p* < 0.05). According to the ROC analysis, the optimal MDW cutoff value with the highest sensitivity and specificity for predicting sepsis in the postoperative period was 20.5. *Conclusions:* Our findings indicate that elevated MDW levels may be a valuable predictor of sepsis in patients following cardiovascular surgery.

## 1. Introduction

The success rate in patients requiring cardiovascular surgery has increased in recent years, mainly due to improvements in diagnostic technology and innovative surgical procedures [1]. Although the proportion of sepsis has decreased by 0.4–4.8% as a result of these advancements, the risk of death remains very high, at 17–79%, if these patients are not identified early [2,3,4]. Thus, early detection and treatment of sepsis are critical. The first two International Consensus Working Groups for Sepsis (Sepsis-1–2) defined sepsis as patients who met systemic inflammatory response syndrome (SIRS) criteria and infections. However, the third meeting (Sepsis-3) stated that the Sequential Organ Failure Assessment (SOFA) score was more useful for sepsis diagnosis rather than SIRS because SIRS can be seen postoperatively, particularly in cardiac surgical patients [4,5,6]. In addition to these criteria, a recent study demonstrated that using inflammatory markers, such as C-reactive protein (CRP), procalcitonin (PCT), and neutrophil-to-lymphocyte ratio (NLR), in the early detection of sepsis was effective [7]. In addition to these traditional parameters, monocyte distribution width (MDW), a new parameter that can be quickly analyzed by a complete blood count (CBC) device, has limited research demonstrating its utility in the diagnosis of sepsis in patients, particularly in emergency departments (EDs) and intensive care units (ICUs) [8,9,10,11,12,13]. However, no research has been conducted on the diagnostic assessment of MDW for the development of sepsis in patients undergoing cardiovascular surgery.

We investigated the diagnostic utility of the MDW parameter for predicting sepsis in patients following cardiovascular surgery.

## 2. Materials and Methods

This cross-sectional study was carried out at the Cardiovascular Surgery Clinic of Gazi University between 2021 and 2022. This study was approved by the Ethics Committee of Gazi University Faculty of Medicine (Approval code and date: 789; 24 October 2022).

### 2.1. Study Groups

This study included 43 consecutive patients who had undergone cardiovascular surgery. All patients were examined at three different time points (TPs): the day before the operation (preoperative at 24 h time point-1) [TP1], at the postoperative 24 h [TP2], and on the day of discharge [TP3]. The major open-heart surgeries performed were as follows: coronary artery bypass grafting (*n* = 33), mitral valve replacement (*n* = 5), aortic valve replacement (*n* = 3), double aortic and mitral valve replacement (*n* = 1), and ascending aorta graft replacement (*n* = 1). Patients with acute hepatitis, venous thrombosis, intestinal bleeding, or advanced chronic obstructive pulmonary disease were also excluded. 

### 2.2. Study Design

Data from all patients during cardiovascular surgery or ICU hospitalization were recorded using the data processing system of our hospital. The third International Consensus Definitions Working Group (Sepsis-3) proposed updated criteria defining sepsis as the presence of clinically and/or microbiologically documented infections and an increase in the SOFA score of more than or equal to 2 in critically ill patients [14]. The SOFA score was assessed at bedside every day before and after surgery by the same physician. The SOFA scoring system was calculated at https://clincalc.com/IcuMortality/SOFA.aspx (accessed date 20 December 2018). In line with this scoring system, clinically apparent infections were recorded as fever and/or a wound, catheter infections, gastroenteritis, pneumonia, and urinary tract infections. Microbiologically documented infections, including positive blood culture, catheter tip culture, urine culture, and endotracheal lavage culture in patients with suspected ventilator-associated pneumonia, were performed to identify microorganisms in this patient. Comorbid risk factors were as follows: hypertension, diabetes mellitus, dyslipidemia, chronic kidney disease, and goiter. According to the institutional policy, patients routinely received sulbactam−ampicillin after open-heart surgery. Based on the clinician’s assessment, empirical broad-spectrum antibiotics were initiated for sepsis suspicion.

### 2.3. Laboratory Parameters

Blood samples were collected from the patients at three different time points. The Unicel^®^ DxH800 (Beckman Coulter, Miami, DL, USA) automatic blood count device was used to examine routine CBC parameters, such as leukocyte counts and NLR, as well as a new research CBC parameter [MDW] in EDTA-K2 blood samples. The MDW reference value was defined according to the Unicel^®^ DxH800 series system manual. NLR was calculated by dividing the neutrophil count by the lymphocyte count. CRP and PCT levels were also analyzed. The serum level of CRP was measured using a nephelometric assay (Specific Protein Analyzer, Beckman, Marburg, Germany) with a normal range of 0 to 6 mg/L. PCT was tested using a Roche Cobas e601^®^ autoanalyzer, Rotkreuz, Switzerland. Its cutoff value was less than 0.5 ng/mL as negative, while PCT is ≥0.5 ng/mL, indicating sepsis. 

### 2.4. Statistical Analysis

The SPSS 24.0 program was used to analyze all variables. The median, range, and mean ± standard deviation are described. The study’s primary endpoint was the predictive value of the MDW for sepsis following cardiovascular surgery. Since this was the first study to evaluate this endpoint and there were no reliable literature data, we estimated the sample size to include enough patients who would develop sepsis in the postoperative period to ensure sufficient power in our results. Accordingly, a sample size estimation based on sepsis prevalence revealed that 43 patients were needed to detect an estimated 3% of sepsis (range: 0.4–4.8% in published open-heart surgery cases [2,3,4] with an error margin of 5% (95% confidence interval (CI)). A post-hoc power analysis was performed on the proportion of patients in a single group, with a sepsis rate of 20% in our study group of 43 patients. In this calculation, we tested whether the determined proportion of 20% in our study group was different from the hypothetical proportion of 3% in the literature [2,3,4]) (H0: *p* = 0.03 versus H1: *p* ≠ 0.03). The comparison was made using a two-sided, one-sample exact test with a Type I error rate (α) of 0.05. To detect a difference (*p*1*-p*0) of 0.17 with a sample size of 43, the power was 0.97462. We performed separate analyses to assess the independent predictive value of MDW and other markers for predicting sepsis. The categorical variables were analyzed using the chi-square test. The Mann−Whitney-U test was performed to compare the independent variables. Wilcoxon test was used to compare the dependent variables. Spearman’s correlation was used to assess the relationship between MDW value and other parameters. We calculated the specificity of the MDW test in accurately identifying patients without sepsis. To assess sensitivity, we evaluated an MDW test’s ability to correctly identify patients with sepsis. The area under the receiver operating characteristic (ROC) curve, which shows true positive (sensitivity) versus false positive rates (1-specificity) as well as positive and negative predictive values, was used to evaluate the ability to distinguish between sepsis and non-sepsis. ROC analysis established the optimal MDW cutoff value for predicting sepsis. The significant *p*-value was less than 0.05.

## 3. Results

The demographic data of the 43 consecutive patients are shown in Table 1. The sex distribution and median age of the study participants were determined.

### 3.1. Demographic Data

Nine (20%) of the 43 patients were diagnosed with sepsis, including SOFA score ≥2.0, and clinically apparent infections using the Sepsis-3 criteria. Three of these patients had microbiologically documented infections. Only one of these three patients (33%) died as a result of confirmed sepsis. These nine patients switched from prophylactic to empirical broad-spectrum antibiotics. The median days of intensive care unit and hospital stay were 3 and 7 days, respectively (Table 1).

### 3.2. Comparison of Leukocyte Count, MDW Value, NLR, CRP, and PCT Levels According to Each TP

At the three different time points, the mean values of leukocyte count, MDW, CRP, PCT, and NLR were significantly higher at TP2 and TP3 than at TP1 (all *p* < 0.05). All these values were significantly higher at TP2 than at TP3 (Table 2).

### 3.3. Comparison of Leukocyte Count, MDW Value, NLR, CRP Level, and PCT Level Based on Sepsis Status

The comorbidity rate was significantly higher in patients with sepsis than those without sepsis. (*p* < 0.05) Patients with sepsis had significantly higher mean values for age, leukocyte count, MDW, CRP, PCT, and NLR than those without sepsis after cardiac surgery (*p* < 0.05) (Table 3)

### 3.4. Correlations of MDW with NLR, CRP, PCT, and SOFA Score

MDW values in patients at TP1 were significantly correlated with CRP (*r* = 0.44) and SOFA scores (*r* = 0.45) (*p* < 0.05). MDW values in patients at TP2 were significantly correlated with NLR (*r* = 0.53), CRP level (*r* = 0.65), PCT level (*r* = 0.74), and SOFA score (*r* = 0.68) (*p* < 0.05). MDW values in patients at TP3 were significantly correlated with NLR (*r* = 0.43), CRP level (*r* = 0.68), PCT level (*r* = 0.72), and SOFA score (*r* = 0.56) (*p* < 0.05).

### 3.5. ROC Analysis

**Figure 1:**
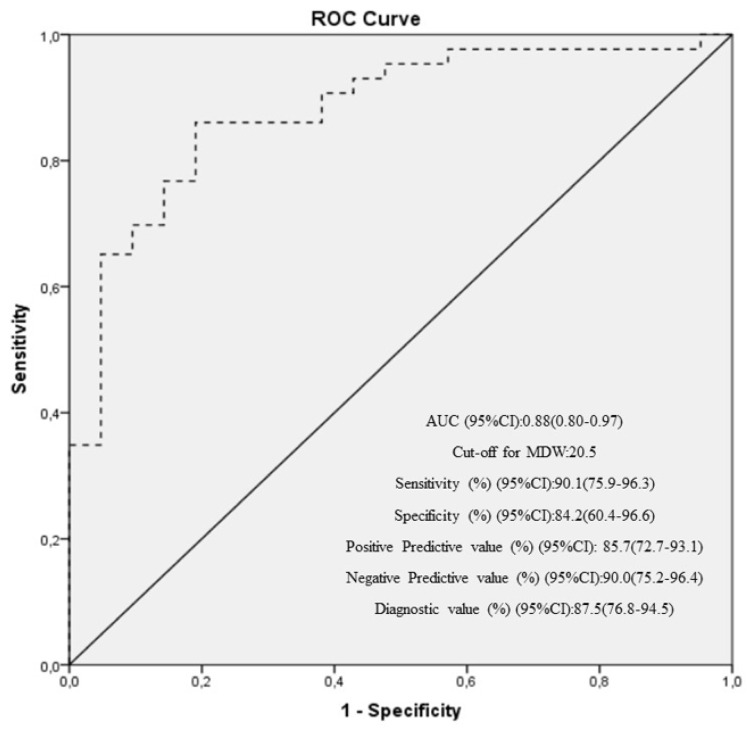
Receiver operating characteristic curve analysis of monocyte distribution width for predicting sepsis after cardiovascular surgery.

The ROC analysis of MDW values for predicting sepsis in patients after cardiovascular surgery is shown in Figure 1. The established area under the curve cutoff values for MDW values for predicting sepsis in the postoperative period was 20.5. In the postoperative period, MDW values were highly sensitive (90.1%; 95% CI 75.9–96.3%) and specific (84.2%; 95% CI 60.4–96.6%) in predicting sepsis. Figure 1 shows positive predictive values of 85.7% (72.7–93.1%) and negative predictive values of 90.0% (75.2–96.4%).

## 4. Discussion

This is the first study to examine the role of MDW in predicting sepsis in patients after cardiovascular surgery. Our key findings are summarized as follows: *i*-postoperative MDW mean values in patients were significantly higher than the preoperative and discharge MDW values. *ii*-the mean value of MDW was significantly higher in patients with sepsis than in those without sepsis. *iii*-SOFA score, CRP, PCT, and NLR were all significantly correlated with postoperative MDW values. *iv*-in patients undergoing cardiovascular surgery, the best cutoff value for MDW in predicting sepsis in the postoperative period was 20.5. 

Postoperative sepsis, although a rare condition following open-heart surgery, is a severe complication that, when it occurs, poses a risk of mortality and leads to prolonged intensive care and hospital stays [15]. In cases of clinical suspicion, prompt diagnosis and initiation of appropriate antibiotic therapy are of the utmost importance for sepsis. Few studies have reported that the range of sepsis and mortality rates in patients undergoing open-heart surgery was 0.4–4.8% and 17–79%, respectively [2,4]. Similarly, we found microbiologically documented sepsis in three (6.9%) of 43 patients, and only one (33%) died as a result of sepsis in our study. 

Recently, there has been an increasing focus on identifying novel biomarkers that provide faster results for the diagnosis of sepsis [16,17]. Indeed, there has been a particular focus on monocytes, which play a crucial role in the initial inflammatory response in both the tissue and systemic circulation in the early stages of sepsis. The shape and distribution of monocytes change rapidly in response to infection [11,18]. These alterations result in a heterogeneous population of monocytes and can be easily detected by the width of monocyte distribution (MDW), which is a reportable parameter of CBC [8,11]. In recent years, an increasing number of studies have reported that MDW, a new parameter of CBC, predicts sepsis, especially in patients treated in the ED and ICU [8,13]. However, there have been no studies on MDW predicting the development of sepsis after cardiac surgery. In metanalyzes reported in numerous retrospective and prospective studies, it was found that the MDW threshold value with high sensitivity and specificity in predicting sepsis in ED and ICU patients was between 19.2 and 24.6 [8]. Similarly, our study’s ROC analysis for elevated MDW values at 24 h postoperatively in predicting sepsis in patients undergoing open-heart surgery provided a cutoff value of 20.5, with 90% sensitivity and 84% specificity. Cardiac surgeons should be alerted to a change in the MDW cutoff value as a “red flag” for suspected sepsis using an automated hematologic analyzer. They should closely observe the patient and perform further investigations to confirm their diagnosis. 

Many investigations have demonstrated that the MDW value measured in K2 EDTA samples was lower than that in K3 EDTA samples; nevertheless, the therapeutic implications for standardizing MDW testing across different settings have not been fully addressed in these studies [8,19]. The effect of the anticoagulant on the MDW value has already been described in the manual of Coulter^®^ instrument’s information (https://www.beckmancoulter.com/en/blog/diagnostics/monocyte-distribution-width) (accessed date: 20 December 2023). As a result, the manufacturer does not recommend utilizing the same cutoff values for K2- or K3-EDTA anticoagulants to avoid false positive/negative results. According to a meta-analysis, one prospective study demonstrated that the MDW cutoff value was 20.5 while using K2 EDTA in the ED [8]. Similarly, we found the same cutoff MDW value for the K2EDTA samples in our analysis. Our findings indicate that using postoperative MDW values for the early detection and identification of sepsis may be valuable for cardiac surgeons; however, they also need to know the type of EDTA samples used for optimal MDW results.

In the Sepsis-3 criteria, a SOFA score ≥2 in addition to proven infection can be used to identify sepsis, especially in patients admitted to the ED or being followed up in the ICU [4]. However, the relationship between SOFA score and MDW value has been reported in a few studies [4,20]. One of these studies indicated that the SOFA score was used in the Sepsis-3 criteria in patients admitted to the ED, 20% of patients using this score were diagnosed with sepsis, and their MDW value was greater than 20 [20]. Based on a meta-analysis, one retrospective study using Sepsis-3 criteria revealed that the sepsis rate was 21.6% and the MDW cutoff value was 19.2 in the ICU [8]. Similarly, we found that nine (20%) of 43 patients undergoing open-heart surgery in the ICU had clinically apparent infections and positive SOFA scores, and their postoperative MDW cutoff was 20.5. In addition, a significant positive correlation between postoperative MDW and SOFA score was observed in our study. Our data show that a postoperative MDW value of 20.5, in addition to the SOFA score, can be utilized to predict sepsis in cardiac surgery patients.

Elevated NLR, CRP, and PCT are well-known traditional biomarkers that have long been used in the suspicion of sepsis [7]. Microbiologically documented infections required to confirm a definitive diagnosis of sepsis can be time consuming. In this context, the NLR cutoff level in patients was determined to be 7.4 for predicting myocardial infarction, 2.6 for predicting preoperative sepsis, and 15 for predicting postoperative sepsis [21,22,23,24,25]. Although no NLR study was found for the diagnosis of sepsis in the postoperative follow-up of patients who underwent cardiovascular surgery, similar results to the published studies in the literature, in our study, mean NLR values in major cardiac surgery patients were 2.1 for preoperative and 14.2 for the postoperative period, respectively. Furthermore, our study demonstrated a moderate to good correlation between postoperative MDW values and CRP, PCT, and NLR, which is consistent with results from previous studies [11,13,18]. In another study, the authors found that patients with infection had significantly higher median values of PCT level and leukocyte counts in the first 10 postoperative days compared to those without infection following cardiac surgery [26]. Similarly, we observed that individuals with sepsis had higher mean MDW, PCT, and leukocyte count values than those without sepsis, following cardiac surgery. Our findings indicate that extended CBC parameters, such as NLR and MDW values, in addition to increased conventional CRP and PCT markers, can be utilized to predict postoperative sepsis in patients undergoing cardiovascular surgery. Thus, adding new parameters to the CBC, such as MDW, will provide cardiac surgeons with an inexpensive and rapid diagnostic marker for predicting sepsis following cardiovascular surgery.

This study had a variety of strengths and limitations. This is the first study to examine the role of MDW in predicting sepsis after cardiovascular surgery. A strength of this study was that extended CBC parameters such as MDW and NLR, in addition to CRP and PCT, were used as readily available and beneficial diagnostic tools for the early detection and identification of sepsis following cardiac surgery. The small sample size and the limited number of patients with sepsis were the major limitations of this study. 

## 5. Conclusions

Our findings suggest that MDW may be a useful predictor of early sepsis detection in patients after cardiovascular surgery; however, further research with a larger sample size is needed.

## Figures and Tables

**Table 1:** Demographic data.

	*n* = 43
Age	
Median, Range	61 (24–88)
Sex, *n*,%	
Male	30 (70%)
Female	13 (30%)
Comorbidity, *n*,%	
Yes	39 (90%)
No	4 (10%)
Sepsis-3 criteria, *n*,%	
-Clinically confirmed infections	
Yes	9 (20%)
No	34 (80%)
-Microbiologically documented infections	
Yes	3 (7%)
No	40 (93%)
Intensive care unit stays (day)	
Median, Range	3 (2–28)
Hospital stays (day)	
Median, Range	7 (4–28)

**Table 2:** Comparison of the mean values of leukocyte count, neutrophil-to-lymphocyte ratio, monocyte distribution width, C-reactive protein, and procalcitonin in patients at three different time points.

Variables (*n* = 43), (Mean ± Standard Deviation)	TP-1	TP-2	TP-3	*p*_1–2_	*p*_1–3_	*p*_2–3_
Leukocyte count (×10^3^/µL)	7.1 ± 2.4	11.8 ± 3.3	9.2 ± 3.6	**0.0001**	**0.0001**	**0.0001**
Neutrophil/lymphocyte ratio	2.5 ± 0.4	15.7 ± 7.2	5.7 ± 0.8	**0.0001**	**0.0001**	**0.0001**
Monocyte distribution width	18.7 ± 2.4	21.6 ± 2.5	19.7 ± 2.3	**0.0001**	**0.01**	**0.001**
C-reactive protein, mg/dL	8.1 ± 1.2	131.5 ± 43.1	105.1 ± 10.1	**0.0001**	**0.0001**	**0.006**
Procalcitonin, ng/mL	0.04 ± 0.01	1.6 ± 0.9	0.3 ± 0.05	**0.0001**	**0.0001**	**0.0001**

TP, time point; TP-1, preoperative period; TP-2, postoperative period at 24 h; TP-3, discharge period.

**Table 3:** Comparison of baseline demographic data and mean values of postoperative leukocyte count, neutrophil-to-lymphocyte ratio, monocyte distribution width, C-reactive protein, and procalcitonin levels based on sepsis status.

		Sepsis		*p*-Value
		Yes *n* = 9	No *n* = 34	
Age (years)	(mean ± SD *)	67.7 ± 11.4	57.8 ± 12.4	0.01
Sex (*n*,%)				
Male		6 (66%)	21 (60%)	0.25
Female		3 (34%)	13 (40%)	
Comorbidity (*n*,%)				0.01
Yes		9 (100%)	20 (62%)	0.006
No		0 (0%)	14 (38%)	
Leukocyte count (×10^3^/µL) (mean ± SD *)		12.4 ± 4.2	10.1 ± 3.5	0.04
Neutrophil/lymphocyte ratio (mean ± SD *)		16.4 ± 6.4	11.1 ± 4.9	0.04
Monocyte distribution width (mean ± SD *)		22.5 ± 3.1	19.6 ± 2.9	0.002
C-reactive protein, mg/dL (mean ± SD *)		139.2 ± 48.1	83.5 ± 16.6	0.008
Procalcitonin, ng/mL (mean ± SD *)		2.1 ± 0.7	0.8 ± 0.2	0.01

* Mean ± standard deviation (SD).

## Data Availability

Data available on request from the authors.

## References

[1] Rahman I.A., Kendall S. (2020). Cardiac surgery in the very elderly: It isn’t all about survival. Br. J. Cardiol..

[2] Reyes D.Á., Piedrahita D.R.E., Flórez M.A. (2021). Sepsis after cardiac surgery: The clinical challenge. Review article. Acta Colomb. Cuid. Intensivo.

[3] de Oliveira D.C., de Oliveira Filho J.B., Silva R.F., Moura S.S., Silva D.J., Egito E.S.T., Piegas L.S. (2010). Sepsis in the Postoperative Period of Cardiac Surgery: Problem Description. Arq. Bras. Cardiol..

[4] Howitt S.H., Herring M., Malagon I., McCollum C.N., Grant S.W. (2018). Incidence and outcomes of sepsis after cardiac surgery as defined by the Sepsis-3 guidelines. Br. J. Anaesth..

[5] Gül F., Arslantaş M.K., Cinel I., Kumar A. (2017). Changing Definitions of Sepsis. Turk. J. Anaesthesiol. Reanim..

[6] Velho T.R., Pereira R.M., Paixão T., Guerra N.C., Ferreira R., Corte-Real H., Nobre Â., Moita L.F. (2022). Sequential Organ Failure Assessment Score in the Intensive Care Unit as a Predictor of Long-Term Survival after Cardiac Surgery. Crit. Care Explor..

[7] Ljungström L., Pernestig A.K., Jacobsson G., Andersson R., Usener B., Tilevik D. (2017). Diagnostic accuracy of procalcitonin, neutrophil-lymphocyte count ratio, C-reactive protein, and lactate in patients with suspected bacterial sepsis. PLoS ONE.

[8] Agnello L., Vidali M., Lo Sasso B., Giglio R.V., Gambino C.M., Scazzone C., Ciaccio A.M., Bivona G., Ciaccio M. (2022). Monocyte distribution width (MDW) as a screening tool for early detecting sepsis: A systematic review and meta-analysis. Clin. Chem. Lab. Med..

[9] Laínez Martínez S., González Del Castillo J. (2022). Usefulness of monocyte distribution width (MDW) as a sepsis biomarker. Rev. Esp. Quimioter..

[10] Huang Y.H., Chen C.J., Shao S.C., Li C.H., Hsiao C.H., Niu K.Y., Yen C.C. (2023). Comparison of the Diagnostic Accuracies of Monocyte Distribution Width, PCT, and C-Reactive Protein for Sepsis: A Systematic Review and Meta-Analysis. Crit. Care Med..

[11] Wu J., Li L., Luo J. (2022). Diagnostic and Prognostic Value of Monocyte Distribution Width in Sepsis. J. Inflamm. Res..

[12] Ciaccio A.M., Agnello L., Sasso B.L., Giglio R.V., Iacona A., Gambino C.M., Scazzone C., Tuttolomondo A., Ciaccio M. (2023). Monocyte Distribution Width (MDW) as a biomarker of sepsis: An evidenced-based laboratory medicine approach. Clin. Chim. Acta.

[13] Meraj F., Shaikh S., Maqsood S., Kanani F., Khan H., Jamal S. (2023). Monocyte Distribution Width, a Novel Biomarker for Early Sepsis Screening and Comparison with PCT and C-Reactive Protein. J. Lab. Physicians.

[14] Vincent J.L., Moreno R., Takala J., Willatts S., De Mendonça A., Bruining H., Reinhart C.K., Suter P.M., Thijs L.G. (1996). The SOFA (Sepsis-related Organ Failure Assessment) score to describe organ dysfunction/failure. On behalf of the Working Group on Sepsis-Related Problems of the European Society of Intensive Care Medicine. Intensive Care Med..

[15] Chen L.F., Arduino J.M., Sheng S. (2012). Epidemiology and outcome of major postoperative infections following cardiac surgery: Risk factors and impact of pathogen type. Am. J. Infect. Control.

[16] Crouser E.D., Parrillo J.E., Seymour C., Angus D.C., Bicking K., Tejidor L., Magari R., Careaga D., Williams J., Closser D.R. (2017). Improved Early Detection of Sepsis in the ED with a Novel Monocyte Distribution Width Biomarker. Chest.

[17] Kim M.H., Choi J.H. (2020). An Update on Sepsis Biomarkers. Infect. Chemother..

[18] Wang S.Y., Mak K.L., Chen L.Y., Chou M.P., Ho C.K. (1992). Heterogeneity of human blood monocyte: Two subpopulations with different sizes, phenotypes and functions. Immunology.

[19] Agnello L., Bivona G., Vidali M., Scazzone C., Giglio R.V., Iacolino G., Iacona A., Mancuso S., Ciaccio A.M., Lo Sasso B. (2020). Monocyte distribution width (MDW) as a screening tool for sepsis in the Emergency Department. Clin. Chem. Lab. Med..

[20] Crouser E.D., Parrillo J.E., Seymour C.W., Angus D.C., Bicking K., Esguerra V.G., Peck-Palmer O.M., Magari R.T., Julian M.W., Kleven J.M. (2019). Monocyte Distribution Width: A Novel Indicator of Sepsis-2 and Sepsis-3 in High-Risk Emergency Department Patients. Crit. Care Med..

[21] Crouser E.D., Parrillo J.E., Martin G.S., Huang D.T., Hausfater P., Grigorov I., Careaga D., Osborn T., Hasan M., Tejidor L. (2020). Monocyte distribution width enhances early sepsis detection in the emergency department beyond SIRS and qSOFA. J. Intensive Care.

[22] Sawant A.C., Adhikari P., Narra S.R., Srivatsa S.S., Mills P.K., Srivatsa S.S. (2014). Neutrophil to lymphocyte ratio predicts short- and long-term mortality following revascularization therapy for ST elevation myocardial infarction. Cardiol. J..

[23] Silberman S., Abu-Yunis U., Tauber R., Shavit L., Grenader T., Fink D., Bitran D., Merin O. (2018). Neutrophil-Lymphocyte Ratio: Prognostic Impact in Heart Surgery. Early Outcomes Late Survival. Ann. Thorac. Surg..

[24] Sarı R., Karakurt Z., Ay M., Çelik M.E., Yalaz Tekan Ü., Çiyiltepe F., Kargın F., Saltürk C., Yazıcıoğlu Moçin Ö., Güngör G. (2019). Neutrophil to lymphocyte ratio as a predictor of treatment response and mortality in septic shock patients in the intensive care unit. Turk. J. Med. Sci..

[25] Wheatley J., Liu Z., Loth J., Plummer M.P., Penny-Dimri J.C., Segal R., Smith J., Perry L.A. (2023). The prognostic value of elevated neutrophil-lymphocyte ratio for cardiac surgery-associated acute kidney injury: A systematic review and meta-analysis. Acta Anaesthesiol. Scand..

[26] Heredia-Rodríguez M., Bustamante-Munguira J., Lorenzo M., Gómez-Sánchez E., Álvarez F.J., Fierro I., Conejo E., Tamayo E. (2017). Procalcitonin and white blood cells, combined predictors of infection in cardiac surgery patients. J. Surg. Res..

